# Triple A-Site Cation Ordering in the Ferrimagnetic Y_2_CuGaMn_4_O_12_ Perovskite

**DOI:** 10.1021/acs.inorgchem.2c02343

**Published:** 2022-08-31

**Authors:** Alexei A. Belik, Dmitry D. Khalyavin, Yoshitaka Matsushita, Kazunari Yamaura

**Affiliations:** † International Center for Materials Nanoarchitectonics (WPI-MANA), National Institute for Materials Science (NIMS), Namiki 1-1, Tsukuba, Ibaraki 305-0044, Japan; ‡ ISIS Facility, Rutherford Appleton Laboratory, Chilton, Didcot OX11 0QX, United Kingdom; § National Institute for Materials Science (NIMS), Sengen 1-2-1, Tsukuba, Ibaraki 305-0047, Japan; ∥ Graduate School of Chemical Sciences and Engineering, Hokkaido University, North 10 West 8, Kita-ku, Sapporo, Hokkaido 060-0810, Japan

## Abstract

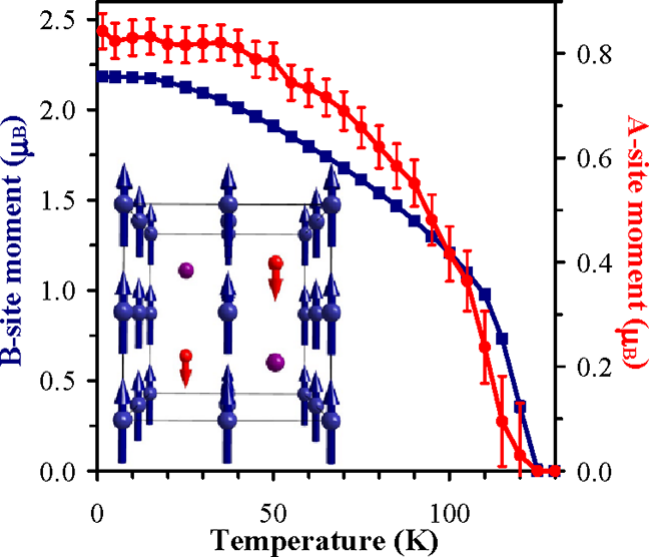

A new member of A-site columnar-ordered A_2_A′A″B_4_O_12_ quadruple perovskites with the composition of Y_2_CuGaMn_4_O_12_ was prepared by a high-pressure, high-temperature method at 6 GPa and about 1500 K. Its crystal structure and cation distributions were studied by powder synchrotron X-ray and neutron diffraction. There is a triple A-site cation ordering with some degrees of anti-site disorder among sites occupied by 3d transition metals: [Y_2_]_A_[Cu_0.8_Mn_0.2_]_A′_[Ga_0.8_Mn_0.2_]_A″_[Mn_3.6_Cu_0.2_Ga_0.2_]_B_O_12_. It has the space group *P*4_2_/*nmc* (no. 137) between 1.5 and 873 K with *a* = 7.33884 Å and *c* = 7.66251 Å at 297 K. Despite anti-site disorder, it exhibits a long-range ferrimagnetic order at *T*_C_ = 115 K with the ordered moment of 2.19 μ_B_ at each B site and 0.89 μ_B_ at the A′ or A″ site. Magnetic moments are aligned along the *c* axis; all moments are ordered ferromagnetically at the B sites, and the moments at the A′ or A″ site are ordered in the opposite direction. Cu^2+^ doping drastically changes magnetic properties as “parent” Y_2_MnGaMn_4_O_12_ just shows spin-glass magnetic properties without long-range ordering. Anisotropic thermal expansion was observed in Y_2_CuGaMn_4_O_12_: the lattice parameter *a* almost linearly decreases from 1.5 K to *T*_C_ and then monotonically increases up to 873 K (almost linearly from 300 K); the parameter *c* monotonically increases from 1.5 to 300 K and then decreases up to 600 K.

## Abstract 2

A new member of the A-site columnar-ordered quadruple perovskite A_2_A′A″B_4_O_12_ family, Y_2_CuGaMn_4_O_12_, was prepared at high pressure and high temperature, with triple A-site ordering and ferrimagnetic properties.

## 1 Introduction

Cation ordering in ABO_3_ perovskites has significant effects on their properties. The main driving forces of cation ordering are differences in cation sizes, oxidation states, and electronic structures.^1,2^ In the case of B-site ordering, B cations have basically the same octahedral coordination. Therefore, oxidation state differences (and resulting differences in cation sizes) become the primary driving force. In the case of B cations, the charge difference can reach as high as 6 (e.g., for Li^+^ and Re^7+^). For example, cation ordering has significant effects on structural, magnetic, and magnetoresistance properties of Sr_2_FeMoO_6_.^3−5^

For A cations, the charge difference does not exceed 3 (e.g., for Na^+^ and Th^4+^), but in most cases, it is just 2 (e.g., for Li^+^/Na^+^ and R^3+^, where R is a rare-earth element) or 1 (e.g., for Ca^2+^/Sr^2+^ and R^3+^). Therefore, the number of examples of the oxidation-state-driven A-site cation ordering is significantly smaller than the number of examples of such B-site cation ordering.^1,2^ In most cases, layered A-site cation ordering is realized when one of the A cations is Li^+^ or even a vacancy.^1^ In some cases, layered A-site cation ordering is promoted by rock-salt B-site cation ordering (e.g., in the so-called doubly ordered NaLaMgWO_6_).^2,6^

In perovskites (if we consider just oxides and fully oxygen stoichiometric ones), the coordination environment of B cations is fixed to the octahedral one. On the other hand, coordination environments of A cations are more flexible. The coordination number of A cations can change from 12 to 7 even for the usually observed *a*^+^*b*^–^*b*^–^ octahedral tilt (the so-called GdFeO_3_-type structure) in the Glazer notation.^7^ The coordination environments of A cations can change drastically for large tilts of the *a*^+^*a*^+^*a*^+^ and *a*^+^*a*^+^*c*^–^ types. The *a*^+^*a*^+^*a*^+^ system with large tilts produces A-site-ordered quadruple perovskites, AA′_3_B_4_O_12_,^8−10^ where the A′ site has a square-planar coordination as the first coordination sphere with eight much longer A′–O distances. The *a*^+^*a*^+^*c*^–^ system with large tilts produces A-site columnar-ordered quadruple perovskites, A_2_A′A″B_4_O_12_,^11^ where the A′ site has a square-planar coordination (similar to AA′_3_B_4_O_12_) and the A″ site has a tetrahedral coordination as the first coordination sphere. Therefore, when the electronic structures of the A, A′, and A″ cations are different, A-site quadruple perovskites can be stabilized even for cations with the same oxidation states (e.g., for Ca^2+^/Sr^2+^ and Cu^2+^/Mn^2+^ and for R^3+^/Bi^3+^ and Mn^3+^). Different electronic structures mean that some cations (namely, at the A′ site) show a very strong tendency for the first-order Jahn–Teller effect and square-planar coordination.

The AA′_3_B_4_O_12_ perovskites are intrinsically fully ordered at the A-sites. Also, the number of examples of the AA′_3_B_4_O_12_ perovskites could even be larger than the number of examples of fully ordered double A_2_BB′O_6_ perovskites, even though it is generally believed that B-site ordering is more common in the latter.^1,12^ The coordination environments of the A_2_A′A″B_4_O_12_ perovskites suggest that they have intrinsic triple A-site ordering. However, the triple ordering by different cations has been experimentally realized just in few examples so far, in R_2_MnGa(Mn_4–*x*_Ga_*x*_)O_12_ (R = Ho and Y) solid solutions,^13,14^ R_2_CuMnMn_4_O_12_ (R = Dy and Y),^13,15^ and Ca(Mn_0.5_Cu_0.5_)FeReO_6_.^16^ In few cases of Sm_2_MnMn(Mn_3_Ti)O_12_ and RMn_3_O_6_,^17,18^ the A′ and A″ sites are occupied by the same element in different oxidation states (e.g., Mn^3+^ at A′ and Mn^2+^ at A″). In the majority of cases, the A′ and A″ sites are occupied by the same cation (e.g., Mn^2+^).^17,19−21^

In this work, we could prepare a new A-site columnar-ordered quadruple perovskite, Y_2_CuGaMn_4_O_12_, where the A, A′, and A″ sites are occupied by different cations, or in other words, with triple A-site ordering. This was achieved by selecting cations (Y^3+^/Cu^2+^/Ga^3+^) with different electronic properties (in other words, with different site preferences). Y_2_CuGaMn_4_O_12_ demonstrates completely different magnetic properties in comparison with the “parent” Y_2_MnGaMn_4_O_12_, which shows spin-glass magnetic properties.^14^ Ferromagnetic (FM) ordering at the B sites and antiparallel ordering at the A′ or A″ sites take place in Y_2_CuGaMn_4_O_12_ at *T*_C_ = 115 K.

## 2 Experimental Section

Y_2_CuGaMn_4_O_12_ was prepared from a stoichiometric mixture of *h*-YMnO_3_, Mn_2_O_3_, Ga_2_O_3_ (99.9%), CuO (99.9%), and MnO_1.839_ (99.995%) at 6 GPa and about 1500 K for 2 h in Au capsules. After annealing at 1500 K, the samples were quenched to room temperature (RT) by turning off the heating current, and pressure was slowly released. Single-phase Mn_2_O_3_ was prepared from commercial “MnO_2_“ (99.99%; with carbonate impurities) by heating in air at 923 K for 24 h. MnO_1.839_ was (another) commercial “MnO_2_“, whose oxygen content was determined by the thermal analysis and which did not contain any carbonate impurities. Single-phase *h*-YMnO_3_ was prepared from a stoichiometric mixture of Y_2_O_3_ (99.9%) and Mn_2_O_3_ by annealing in air at 1430 K for 60 h with several intermediate grindings. The use of *h*-YMnO_3_ (instead of Y_2_O_3_) allowed increasing the sample amount prepared in one high-pressure high-temperature experiment.

X-ray powder diffraction (XRPD) data were collected at RT on a Rigaku MiniFlex600 diffractometer using Cu Kα radiation (2θ range of 8–100°, a step width of 0.02°, and a scan speed of 2°/min). High-temperature XRPD data were measured in air on a Rigaku SmartLab instrument (Cu Kα_1_ radiation at 45 kV and 200 mA; 2θ range of 15–105°, a step width of 0.02°, and a scan speed of 1°/min) from 293 to 873 K and from 873 to 293 K with a step of 20 K (Bragg–Brentano geometry was used for all laboratory XRPD). Synchrotron XRPD data were collected from 100 to 850 K (a heating rate was 30 K/min between temperature points; waiting time was 120 s at 100 K and 10 s at all other temperatures) and then at 297 K (without cooling/hearing guns) on the beamline BL02B2 of SPring-8 (the intensity data were taken between 2.08 and 78.22° at 0.006° intervals in 2θ using a wavelength of λ = 0.413854 Å; the data between 2.08 and 60.0° were used in the structural analysis as no experimental reflections were observed above 60.0°).^22^ High statistics data with the measurement time of 300 s were collected at 100, 297, and 700 K; at all other temperatures, the measurement time was 10 s (note that the total measurement time was 600 and 20 s, respectively, due to one movement of detectors). The sample was placed into an open Lindemann glass capillary tube (inner diameter: 0.2 mm), which was rotated during measurements. The Rietveld analysis of all XRPD data was performed using the RIETAN-2000 program.^23^

Neutron powder diffraction data were collected at the ISIS-pulsed neutron and muon spallation sources at the Rutherford Appleton Laboratory (UK) using the WISH diffractometer located at the second target station.^24^ The sample (with a total weight of about 1.31 g from three capsules) was loaded into a cylindrical 3 mm diameter vanadium can and measured at 1.5 K from 5 to 150 K with a step of 5 K and at 200 K. The Rietveld analysis of neutron data was performed using the *FullProf* program^25^ against the data measured in detector banks at average 2θ values of 58, 90, 122, and 154°, each covering 32° of the scattering plane.

Magnetic measurements were performed on SQUID magnetometers (Quantum Design, MPMS-XL-7T and MPMS3) between 2 and 400 K in applied fields of 100 Oe and 10 kOe under both zero-field-cooled (ZFC) and field-cooled on cooling (FCC) conditions. Isothermal magnetization measurements were performed between −70 and 70 kOe at different temperatures. The specific heat *C*_p_ at magnetic fields of 0 Oe and 90 kOe was recorded from 300 to 2 K by a pulse relaxation method using a commercial calorimeter (Quantum Design PPMS).

Scanning electron microscopy (SEM) images of fractured surfaces of pellets were obtained on a Hitachi Miniscope TM3000 (operating at 15 kV) (Figure S1a); energy-dispersive X-ray (EDX) spectra were taken on the same instrument (Figure S1b). The cation ratio was 2:1:1:4 for Y/Cu/Ga/Mn within 2σ.

## 3 Results and Discussion

The synthesized Y_2_CuGaMn_4_O_12_ samples were single-phase within the sensitivity of the neutron and synchrotron XRPD data. Note that a small amount of Au impurity was observed in the synchrotron XRPD data, but the Au impurity appeared due to contamination from Au capsules. Therefore, the total chemical composition was constrained to the nominal one during the structural analysis. Y_2_CuGaMn_4_O_12_ was found to crystallize in the space group *P*4_2_/*nmc* at all temperatures from 1.5 to 873 K. In other words, Y_2_CuGaMn_4_O_12_ adopts the parent structure of A-site columnar-ordered quadruple perovskites, A_2_A′A″B_4_O_12_.^11^

The introduction of Mn^4+^ cations (in the form of MnO_2−δ_) into starting oxide mixtures containing R_2_O_3_ and Mn_2_O_3_ often resulted in the formation of the RMn_2_O_5_ impurity during the high-pressure synthesis of such perovskites.^18^ However in the case of Y_2_CuGaMn_4_O_12_, the formation of the YMn_2_O_5_ impurity was suppressed, probably due to a specific combination of other cations.

X-ray scattering factors of Cu^2+^ (27 electrons) and Ga^3+^ (28 electrons) are nearly the same. The same is true for coherent neutron scattering lengths, 7.718 fm for Cu and 7.288 for Ga. Therefore, it is very difficult to distinguish Cu and Ga (by non-resonant X-ray diffraction) and refine their distribution at one site, even with neutron diffraction. However, considering that the A′ and A″ sites in A_2_A′A″B_4_O_12_ perovskites are fundamentally different, we can assume that Ga^3+^ cations cannot occupy the square-planar A′ site and would strongly prefer the tetrahedral A″ site.^26^ On the other hand, Cu^2+^ cations would strongly prefer a square-planar site instead of a tetrahedral site.^26^ X-ray scattering factors of Mn differ enough from those of Cu^2+^ and Ga^3+^ to check cation distributions from synchrotron XRPD data. The existence of anti-site disorder could be seen from reduced occupation factors of the Cu and Ga sites if refined: *g*(Cu) = 0.943(7) and *g*(Ga) = 0.969(7) (the values obtained at 100 K are reported). The occupation factor of the Mn site was just slightly higher than 1: *g*(Mn) = 1.017(3). The anti-site disorder was refined assuming the full occupation of the cation sites (i.e., the total occupation factor was 1). The cation distributions were refined to 0.72(4)Cu + 0.28 Mn for the A′ site and 0.90(3)Ga + 0.10 Mn for the A″ site at 100 K, 0.72(3)Cu + 0.28 Mn and 0.90(3)Ga + 0.10 Mn at 297 K, and 0.72(4)Cu + 0.28 Mn and 0.87(3)Ga + 0.13 Mn at 700 K from the synchrotron data (note that all structural and non-structural parameters were refined simultaneously, including all isotropic atomic displacement parameters). The occupation factor of the Y site was unity within 2σ [e.g., *g*(Y) = 1.004(3) at 100 K].

**Figure 1:**
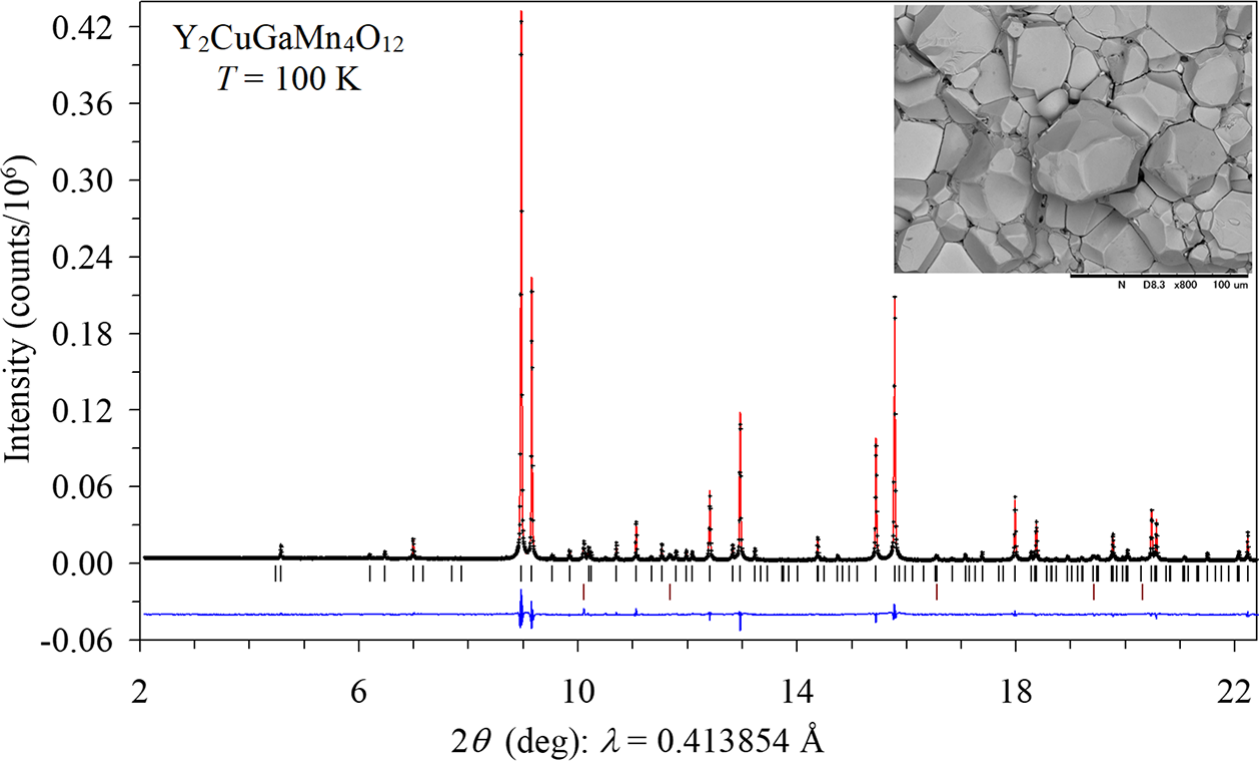
Experimental (black crosses), calculated (red line), and difference (blue line at the bottom) synchrotron powder X-ray diffraction patterns of Y_2_CuGaMn_4_O_12_ at *T* = 100 K in the 2θ range of 2 and 22.4°. The tick marks show possible Bragg reflection positions for Y_2_CuGaMn_4_O_12_ (the first row) and Au impurity (the second row; contamination from Au capsules). Inset shows a typical SEM image (at RT) of the fractured surface of the as-synthesized sample; the scale bar is 100 μm.

**Figure 2:**
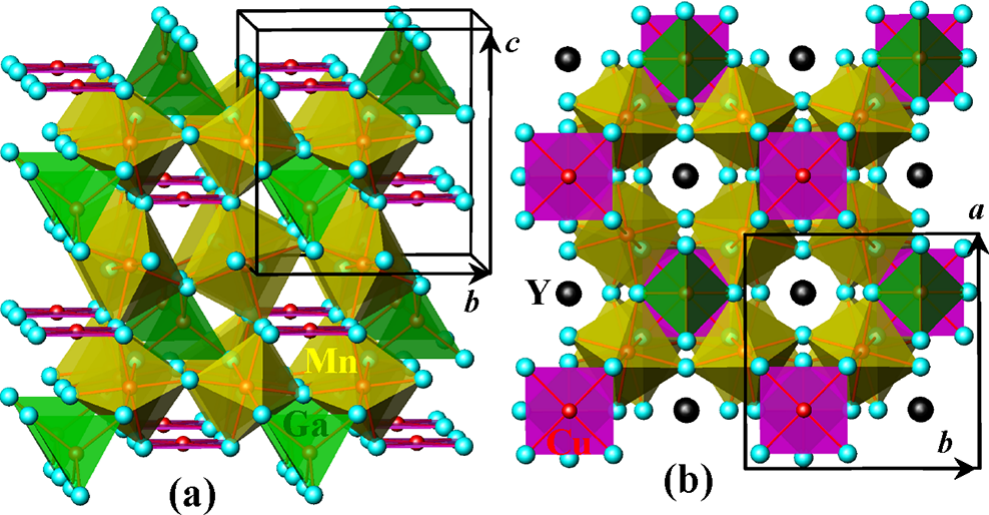
Crystal structure of Y_2_CuGaMn_4_O_12_ in a polyhedral presentation for magnetic cations with CuO_4_ square-planar units (magenta), GaO_4_ tetrahedra (green), and MnO_6_ octahedra (yellow) viewed (a) along the *a* axis and (b) along the *c* axis. Y atoms were omitted on panel (a) for clarity, and they are shown by black spheres on panel (b).

The neutron scattering length of Mn (−3.73 fm) differs significantly from that of Cu and Ga. Therefore, neutron diffraction can give stronger evidence about anti-site disorder (e.g., the refined occupation factors were noticeably smaller than unity for the Cu, Ga, and Mn sites: *g*(Cu) = 0.692(7), *g*(Ga) = 0.688(8), and *g*(Mn) = 0.721(8)) and a more precise distribution of Mn and Cu/Ga atoms. The cation distributions were refined to 0.798(5)Cu + 0.202 Mn for the A′ site and 0.800(5)Ga + 0.200 Mn for the A″ site at 200 K using the neutron diffraction data. To keep the total chemical composition, anti-site disorder was assumed with the B site. Therefore, this cation distribution, [Y_2_]_A_[Cu_0.8_Mn_0.2_]_A′_[Ga_0.8_Mn_0.2_]_A″_[Mn_3.6_Cu_0.2_Ga_0.2_]_B_O_12_, was fixed in all other refinements using both synchrotron and neutron diffraction data. Refined structural parameters from neutron diffraction at 200 K, primary bond lengths, and bond-valence sums (BVS)^27^ are summarized in Tables 1 and 2. Refined structural parameters from synchrotron diffraction at 100, 297, and 700 K are given in Table S1. Experimental, calculated, and difference synchrotron patterns of Y_2_CuGaMn_4_O_12_ at 100 K are shown in Figure 1 and neutron patterns at 200 K in Figure S2. The crystal structure of Y_2_CuGaMn_4_O_12_ is plotted in Figure 2.

**Table 1:** Structural Parameters of Y_2_CuGaMn_4_O_12_ at 200 and 1.5 K from Neutron Powder Diffraction Data^a^

atom	WP	*x*	*y*	*z*	*U*_iso_ (Å^2^)
*T* = 200 K					
Y (A)	4*d*	0.25	0.25	0.2214(2)	0.0228(6)
Cu (A′)	2*a*	0.75	0.25	0.75	0.0218(19)
Ga (A′′)	2*b*	0.75	0.25	0.25	0.0184(19)
Mn (B)	8*e*	0	0	0	0.0196(11)
O1	8*g*	0.25	0.0683(2)	–0.03578(18)	0.0194(7)
O2	8*g*	0.25	0.5456(2)	0.59338(18)	0.0223(6)
O3	8*f*	0.43653(14)	–*x*	0.25	0.0277(7)
*T* = 1.5 K					
Y	4*d*	0.25	0.25	0.2215(2)	0.0230(5)
Cu	2*a*	0.75	0.25	0.75	0.0232(11)
Ga	2*b*	0.75	0.25	0.25	0.0181(11)
Mn	8*e*	0	0	0	0.0204(8)
O1	8*g*	0.25	0.0685(2)	–0.03588(17)	0.0201(5)
O2	8*g*	0.25	0.5461(2)	0.59340(17)	0.0225(5)
O3	8*f*	0.43645(14)	–*x*	0.25	0.0273(5)
M(A′ or A′′)		–0.89(3)μ_B_			
M(B)		2.19(1)μ_B_			

a Source: time-of-flight neutron diffraction; *d*-space range: 0.43–50.0 Å. Crystal system: tetragonal; Space group: *P*4_2_/*nmc* (no. 137, cell choice 2); *Z* = 2. Molecular weight: 722.8258 g/mol. 200 K: *a* = 7.33021(6) Å, *c* = 7.66245(9) Å, and *V* = 411.719(7) Å^3^; *R*_wp_ = 4.72%, *R*_p_ = 4.59%, *R*_I_ = 5.74%, and *R*_wp_exp_ = 1.52%. 1.5 K: *a* = 7.32945(6) Å, *c* = 7.65524(9) Å, and *V* = 411.246(7) Å^3^; *R*_wp_ = 5.01%, *R*_p_ = 4.49%, *R*_I_ = 5.62%, and *R*_wp_exp_ = 0.70%; *R*_Bragg,mag_ = 2.37%. *g*(Y) = *g*(O1) = *g*(O2) = *g*(O3) = 1, where *g* is the occupation factor. Occupation factors (refined at 200 K; fixed at 1.5 K): *g* = 0.798(5)Cu + 0.202 Mn for the Cu site, *g* = 0.800(5)Ga + 0.200 Mn for the Ga site, *g* = 0.8995 Mn + 0.0505Cu + 0.05Ga for the Mn site.

**Table 2:** Bond Lengths (in Å; Below 3 Å), Bond Angles (in deg), Bond-Valence Sum, and Distortion Parameters of MnO_6_ (Δ) in Y_2_CuGaMn_4_O_12_ at 1.5 and 200 K

	1.5 K	200 K
Y–O1 ×2	2.2846(18)	2.2882(19)
Y–O1 ×2	2.3773(19)	2.3785(19)
Y–O2 ×2	2.3815(16)	2.3785(16)
Y–O3 ×4	2.6827(10)	2.6828(16)
BVS(Y^3+^)	+3.11	+3.10
Cu–O3 ×4	1.9326(10)	1.9337(10)
BVS(Cu^2+^)	+2.02	+2.01
Ga–O2 ×4	1.9159(14)	1.9197(14)
Ga–O1 ×4	2.8524(16)	2.8528(15)
BVS(Ga^3+^)	+2.61	+2.59
Mn–O1 ×2	1.9197(4)	1.9194(4)
Mn–O2 ×2	1.9957(5)	1.9955(6)
Mn–O3 ×2	2.0240(3)	2.0254(3)
Δ(MnO_6_)	4.9 × 10^–4^	5.1 × 10^–4^
BVS(Mn^3+^)	+3.34	+3.33
Mn–O1–Mn ×2	145.31(8)	145.40(8)
Mn–O2–Mn ×2	133.31(7)	133.37(7)
Mn–O3–Mn ×2	142.01(5)	142.09(5)

As it is difficult to apply neutron diffraction for all such perovskites, it was important to understand the sensitivity of X-ray diffraction in studying cation distributions. Our above results showed that even high-quality synchrotron XRPD can only give qualitative information for 3d elements—cation distributions found by XRPD can result in large errors (e.g., reaching 30–100% in our case for the Mn content at the A′ and A″ sites).

**Figure 3:**
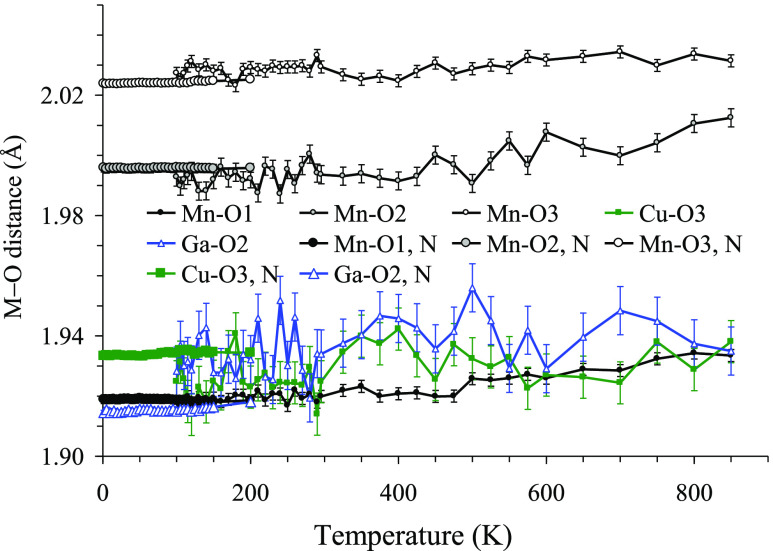
Temperature dependence of the Cu–O3, Ga–O2, Mn–O1, Mn–O2, and Mn–O3 bond distances between 1.5 and 850 K. The distances between 1.5 and 200 K were obtained from neutron diffraction data (large symbols; legends are marked by N; error bars are smaller than the symbols). The distances between 100 and 850 K were obtained from synchrotron X-ray diffraction data (small symbols with error bars).

Figure 3 gives temperature dependence of the main bond lengths of the A′, A″, and B sites. Neutron diffraction gives more accurate information about the positions of the oxygen atoms and less noisy data. Nevertheless, Figure 3 illustrates that the Cu–O3, Ga–O2, Mn–O1, Mn–O2, and Mn–O3 distances remain nearly temperature independent between 1.5 and 850 K. At first glance, the MnO_6_ octahedron appears as a Jahn–Teller distorted one with an unusual −Q_3_ distortion^1^ (where two Mn–O bonds become shorter and four Mn–O bonds become longer in comparison with an undistorted MnO_6_ octahedron – this kind of distortion is realized in CaMn_7_O_12_ at RT)^28^ as the Mn–O1 distance is much shorter than the Mn–O2 and Mn–O3 distances. However, the analysis of symmetry-adapted atomic displacive modes^29,30^ showed that none of them individually can be associated with the cooperative Jahn–Teller distortion. This implies that the above-mentioned anisotropy of the MnO_6_ octahedra is an accidental effect of the superposition of several distortion modes unrelated directly to the lifting of degeneracy between the *e*_g_ electrons of Mn^3+^.

The BVS values (at 200 K) of +3.10 for the Y site were close to the ideal value of +3. The BVS values of +2.01 for the Cu site were also close to the ideal value of +2. The BVS values of +3.33 for the Mn site were close to the average oxidation state of +3.25 for cations at this site. On the other hand, the BVS values of +2.60 for the Ga site noticeably deviated from the expected value of +3. The existence of anti-site disorder can explain such an underbonded value. The oxidization state of manganese at the tetrahedral Ga site should be +2 as Jahn–Teller active Mn^3+^ cations are not found in tetrahedral sites.^26^ However, the ionic radius of Mn^2+^ cations (r_IV_(Mn^2+^) = 0.66 Å) is noticeably larger than that of Ga^3+^ cations (r_IV_(Ga^3+^) = 0.47 Å).^31^ Therefore, the presence of Mn^2+^ cations increases the observed Ga–O bond lengths and decreases the resulting BVS values.

**Figure 4:**
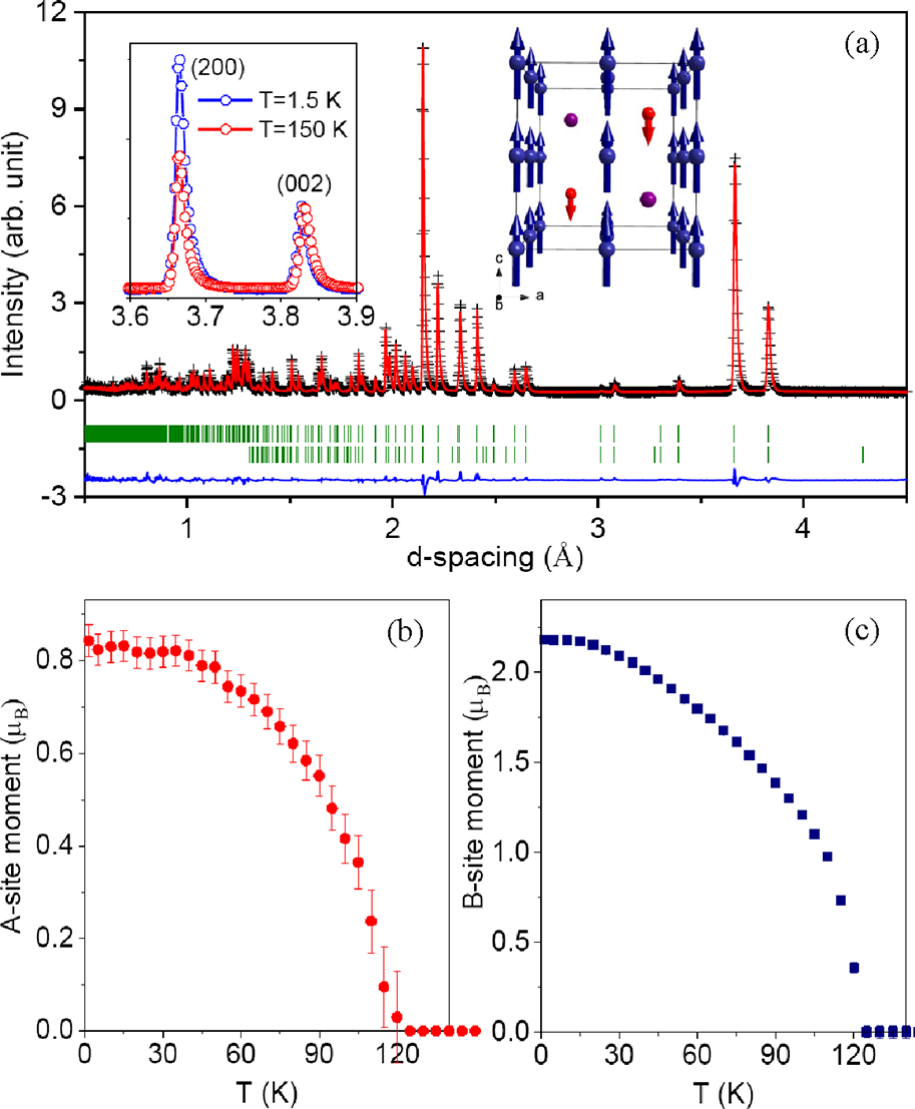
(a) Experimental (black crosses), calculated (red line), and difference (blue line at the bottom) neutron powder diffraction patterns of Y_2_CuGaMn_4_O_12_ at *T* = 1.5 K. The tick marks show possible Bragg reflection positions for the nuclear structure (the first row) and the magnetic structure (the second row). The left inset compares intensities of the (200) and (002) peaks at *T* = 1.5 K (blue circles) and 150 K (red circles). The right inset shows the resulting magnetic structure. The structure implies the #137.513 *P*4_2_/*nm*′*c*′ magnetic symmetry, which keeps the lattice vectors and origin of the paramagnetic space group. (b) Temperature dependence of the ordered magnetic moments on the A′ or A″ site. (c) Temperature dependence of the ordered magnetic moments on the B site.

Cooling below *T*_C_ = 115 K resulted in a significant increase in intensity for some of the diffraction peaks in neutron diffraction patterns (Figure 4a, left insert). This observation indicates an onset of long-range magnetic ordering with propagation vector ***k*** = 0. The absence of magnetic scattering to the (00*l*) reflections points to a simple collinear magnetic order with the spins polarized along the *c*-axis. Indeed, refinement of the magnetic structure assuming three collinear sublattices for A′, A″, and B sites provided a very good fitting quality. The refinement yields the moment size of 2.19(1)μ_B_ for the B site and −0.89(3)μ_B_ and −0.05(3)μ_B_ for the A′ and/or A′′ sites. The latter two values cannot be unambiguously assigned to the A′ and A″ sublattices either based on the refinement quality or on the obtained site occupancies. Although the A″ site is supposed to be non-magnetic in the fully ordered structure, the presence of a small amount of Mn^2+^ (*S* = 5/2) can result in a finite ∼(5*0.2 = 1μ_B_) moment for this sublattice, polarized by the A–B exchange.

The net magnetization of the A′ sublattice can also be strongly dependent on the sign of the A–B exchange between Cu/Mn in this position and the B-site Mn. If the sign is different for Cu^2+^ and Mn^3+^ in the A′ site, then only a small net moment ∼(4*0.2 – 1*0.8 = 0μ_B_) should be expected and it can be assigned to the nearly zero-moment (0.05μ_B_) sublattice obtained in the refinement procedure. The reduced moment size of the B-site Mn can be naturally attributed to the presence of non-magnetic Ga^3+^ cations as well as magnetic Cu^2+^ with negative Cu–O–Mn superexchange interactions. Experimental, calculated, and difference neutron patterns of Y_2_CuGaMn_4_O_12_ at 1.5 K and the magnetic structure are shown in Figure 4a. Figure 4b,c shows temperature dependence of the ordered magnetic moments on the A′ or A″ site and on the B site, respectively. Refined structural and magnetic parameters from neutron diffraction at 1.5 K, primary bond lengths, and BVS^27^ values are summarized in Tables 1 and 2.

**Figure 5:**
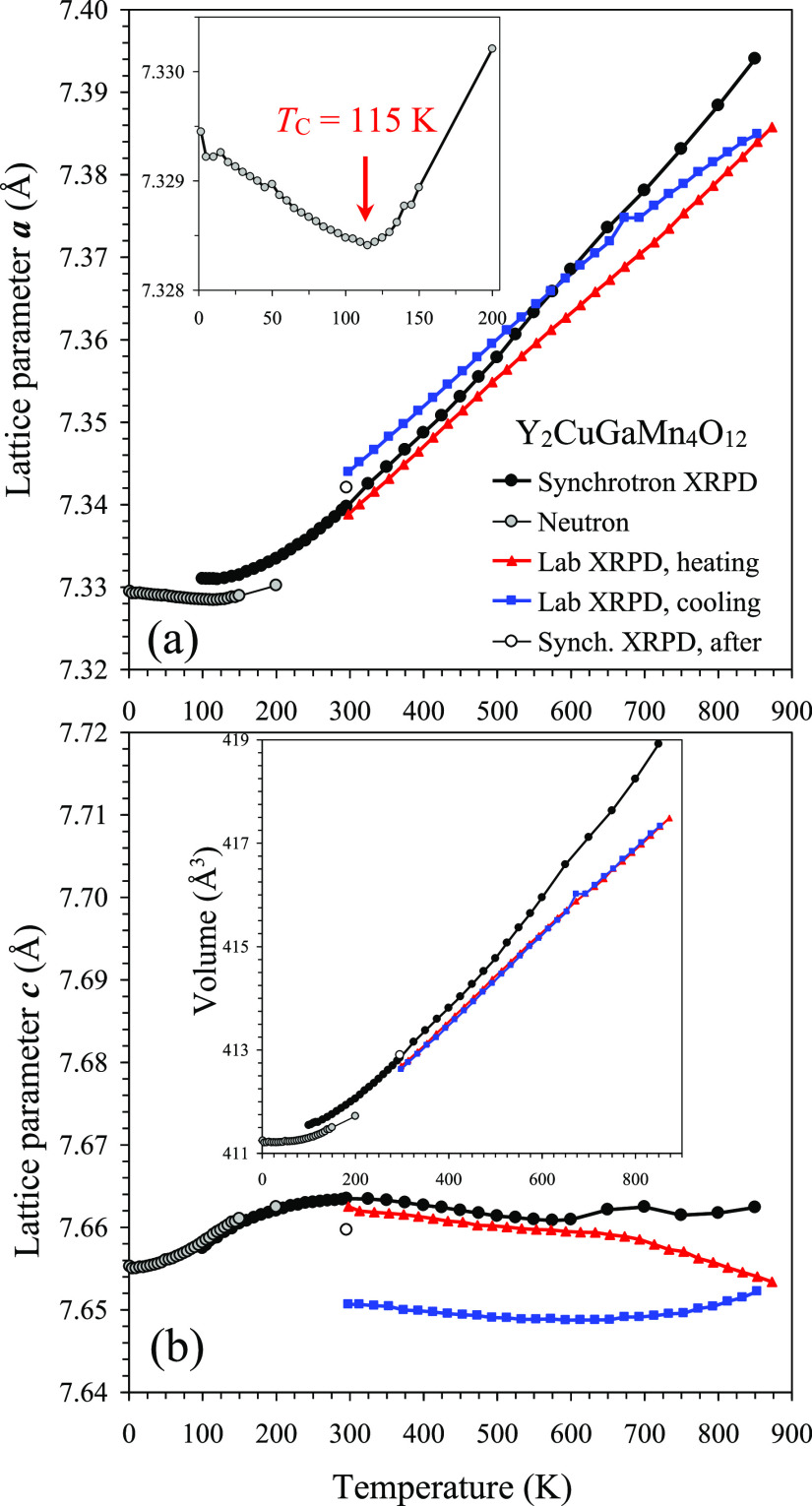
Temperature dependence of the *a* (a) and *c* (b) lattice parameters of Y_2_CuGaMn_4_O_12_ between 1.5 and 873 K. The data from 1.5 to 200 K were obtained from neutron diffraction on heating (gray circles). The data from 100 to 850 K were obtained from synchrotron XRPD on heating (black circles) and then at 297 K (white circles). The data between 293 and 873 K were obtained from laboratory XRPD on heating (red triangles) and cooling (blue squares). Inset in panel (a) shows details from neutron diffraction; the arrow gives the magnetic transition temperature. Inset in panel (b) gives temperature dependence of the unit-cell volume.

Temperature dependence of the lattice parameters in Y_2_CuGaMn_4_O_12_ is shown in Figure 5. There were some differences in the absolute values of the lattice parameters depending on the data set. However, such differences are often observed due to different wavelengths and zero shifts. Moreover, samples from different synthetic batches were used for neutron diffraction and X-ray diffraction—this fact could also contribute to slightly different absolute values. However, the general tendencies are the same. The lattice parameter *a* almost linearly decreases from 1.5 K to *T*_C_ = 115 K and then monotonically increases up to 873 K with almost linear behavior above 300 K. The *c* lattice parameter monotonically increases from 1.5 to 300 K and then gradually decreases up to 600 K. The temperature behavior of parameter *c* above 600 K was slightly different for the synchrotron and laboratory XRPD data sets. Parameter *c* continues to decrease for the laboratory XRPD data set but demonstrates a tiny increase for the synchrotron XRPD data set. This difference could originate from a completely different timescale of the measurements: the synchrotron data above 600 K were collected in about 20 min (with 10 min at 700 K resulting in an additional small drop above 700 K), while the laboratory data above 600 K were collected in about 15 h, where larger changes in the oxygen content or stronger structural relaxation could take place. After heating, the lattice parameters (at RT) showed the same qualitative changes for both the laboratory and synchrotron XRPD data sets—parameter *a* increases and parameter *c* decreases. The absolute values were again different because of different time scales. Such changes in the lattice parameters should be intrinsic and not related to zero shifts, as no changes in zero shift parameters were expected for the synchrotron data set, and the sample height was adjusted at each temperature point for the laboratory data set. Irreversible changes in the lattice parameters during heating and cooling were also observed in another A-site columnar-ordered perovskite, NaSmMn_2_Ti_4_O_12_.^20^

**Figure 6:**
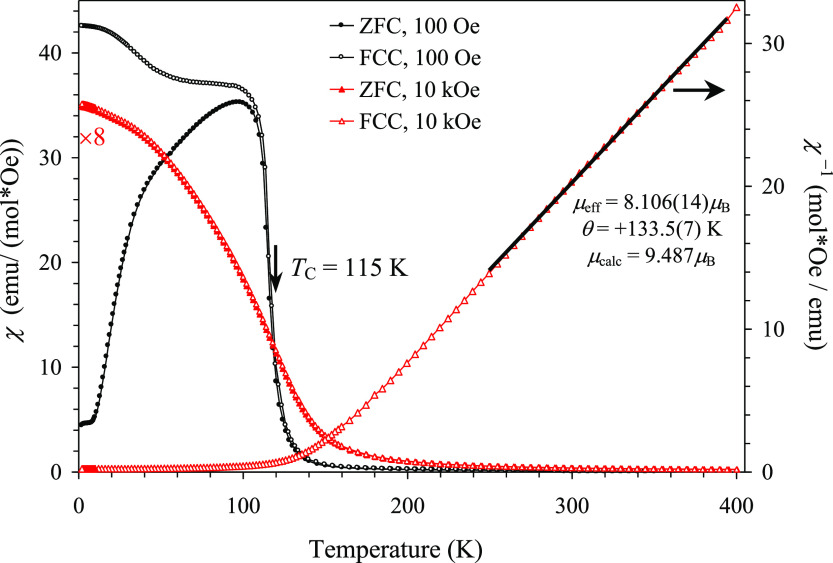
Left–hand axis shows ZFC (filled symbols) and FCC (empty symbols) dc magnetic susceptibility (χ = *M*/*H*) curves of Y_2_CuGaMn_4_O_12_ measured at 100 Oe and 10 kOe (multiplied by 8). Right-hand axis gives the FCC χ^–1^ versus *T* curves at 10 kOe with the Curie–Weiss fit (black line). Parameters of the fit are shown in the figure.

Temperature-dependent magnetic measurements in a small magnetic field of 100 Oe showed sharp rises in magnetic susceptibilities above *T*_C_ = 115 K due to the development of a strong FM moment (Figure 6). A difference between the ZFC and FCC χ versus *T* curves was also observed below about 115 K at *H* = 100 Oe. However, the ZFC and FCC χ versus *T* curves nearly merged at *H* = 10 kOe. The χ^–1^ versus *T* curves follow the Curie–Weiss law (without a temperature-independent term) between about 250 and 400 K. The Curie–Weiss temperature θ was positive [θ = +133.5(7) K], indicating a predominantly FM interaction between magnetic ions. This is consistent with the FM structure at the B sites found from neutron diffraction.

**Figure 7:**
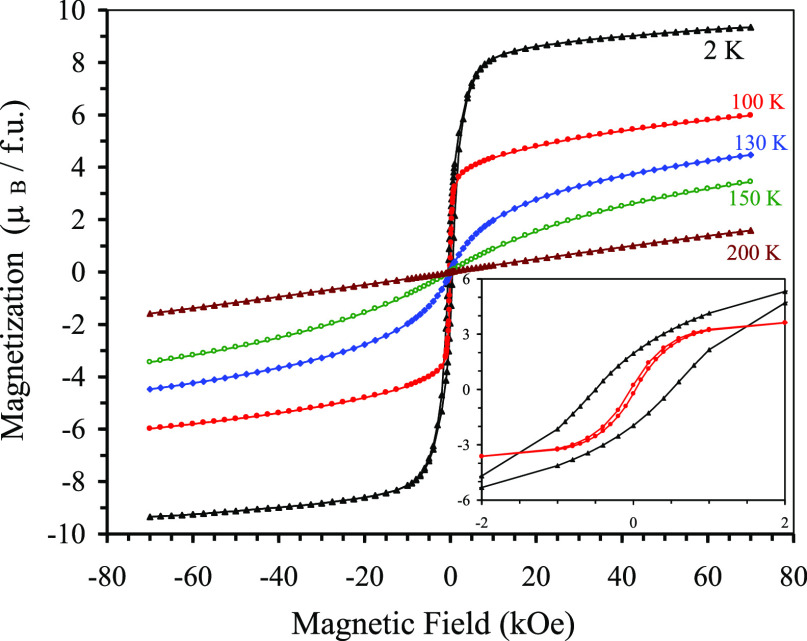
*M* versus *H* curves of Y_2_CuGaMn_4_O_12_ at different temperatures (f.u.: formula unit). Inset shows details near the origin at 2 and 100 K.

**Figure 8:**
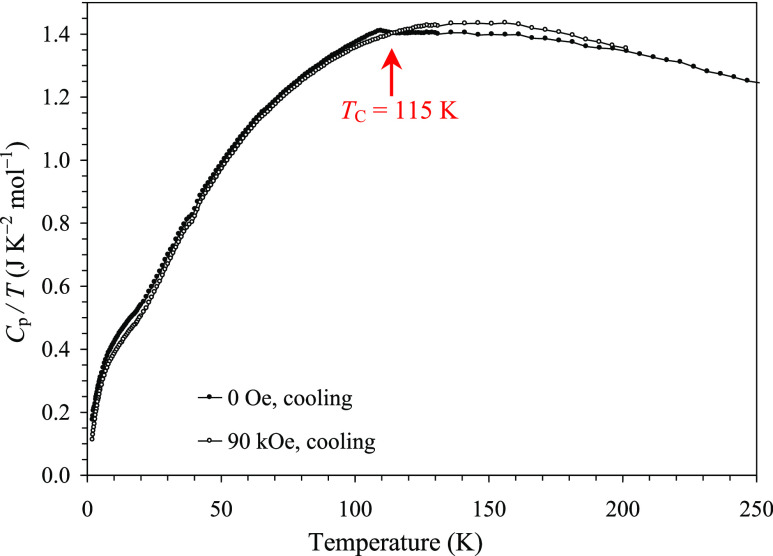
*C*_p_/*T* versus *T* curves of Y_2_CuGaMn_4_O_12_ at *H* = 0 Oe (black filled circles) and 90 kOe (empty circles), where *C*_p_ is the total specific heat. The arrow gives the magnetic transition temperature.

Magnetic-field-dependent magnetic measurements at *T* = 2, 100, 130, 150, and 200 K (Figure 7) showed a behavior typical for soft ferromagnets (or ferrimagnets) with the coercive field *H*_C_ of about 500 Oe at *T* = 2 K and 0 Oe at *T* = 100 K. Magnetization reaches about 9.35μ_B_ at *T* = 2 K and *H* = 70 kOe. This value is close to the full saturation moment of 9.58μ_B_ expected for the FM polarization and using the experimentally determined saturated magnetic moments for the B and A sites. The *M* versus *H* curves had an S-type shape slightly above *T*_C_ (at 130 and 150 K), suggesting the presence of short-range magnetic correlations. Above 200 K, the *M* versus *H* curves were nearly linear, as expected for paramagnetic states. Specific heat measurements (Figure 8) showed a weak anomaly near *T*_C_ = 115 K, confirming a long-range magnetic ordering and a weak additional kink near 20 K. A magnetic field of 90 kOe generally had a weak effect on specific heat, smearing the transition at *T*_C_ = 115 K and moving magnetic entropy to higher temperatures as usually observed in ferromagnets (or ferrimagnets).

The magnetic properties of Y_2_CuGaMn_4_O_12_ are significantly different from those of the “parent” Y_2_MnGaMn_4_O_12_ compound.^14^ No long-range magnetic ordering was found in Y_2_MnGaMn_3_GaO_12_ and Ho_2_MnGaMn_4_O_12_ using neutron diffraction, and only diffuse magnetic scattering was observed.^13,14^ Magnetic measurements also suggested spin-glass-like magnetic properties of Y_2_MnGaMn_4_O_12_.^14^ This behavior was quite unexpected considering the high concentration of magnetic Mn atoms at the B sites. All Mn atoms are formally trivalent in Y_2_MnGaMn_4_O_12_. Cu^2+^ doping introduces Mn^4+^ cations into the B sites, and the double-exchange interaction between Mn^3+^ and Mn^4+^ cations at the B sites could be responsible for an FM structure observed at the B sites of Y_2_CuGaMn_4_O_12_ similar to ferromagnetism observed in La_1–*x*_Ca_*x*_MnO_3_ and La_1–*x*_Sr_*x*_MnO_3_.^32^ However, we note that ferromagnetism was only observed in R_1–*x*_Ca_*x*_MnO_3_ and R_1–*x*_Sr_*x*_MnO_3_ with large R^3+^ cations (e.g., La, Pr, and Nd) and, therefore, large Mn–O–Mn bond angles. In the case of small R^3+^ cations (e.g., Tb–Lu) and smaller Mn–O–Mn bond angles, R_1–*x*_Ca_*x*_MnO_3_ compounds demonstrate spin-glass or antiferromagnetic transitions.^33−38^ Y_2_CuGaMn_4_O_12_ has very small Mn–O–Mn bond angles (Table 2), even smaller than those of Lu_1–*x*_Ca_*x*_MnO_3_ (e.g., 149.0 and 149.7° for *x* = 0.5).^35^ Therefore, the presence of magnetic cations at the A′ and A″ sites in Y_2_CuGaMn_4_O_12_ should also play an important role for the establishment of the FM structure at the B sites. This is consistent with the conclusion by Vibhakar et al.^39^ that the A–B exchange is the key ingredient for understanding magnetic ground states of such columnar-ordered perovskites.

The triple A-site ordering in Y_2_CuGaMn_4_O_12_ was realized through the selection of appropriate cations with different electronic properties. Ga^3+^ has the 3d^10^ configuration, and it strongly prefers tetrahedral and octahedral environments.^26^ Ga^3+^ has never been found in a square-planar coordination. Cu^2+^ has the 3d^9^ configuration, and it shows a very strong tendency for the first-order Jahn–Teller effect and prefers distorted octahedral environments, with extreme cases reaching square-planar and pyramidal coordinations.^26^

## 4 Conclusions

In conclusion, a new member was added to the A-site columnar-ordered quadruple perovskite family with the composition of Y_2_CuGaMn_4_O_12_. Triple A-site cation ordering was achieved by selecting Y^3+^, Cu^2+^, and Ga^3+^ cations with different electronic properties—such a combination had not been reported before. The crystal structure was studied by synchrotron powder X-ray diffraction and neutron diffraction. A small anti-site disorder was observed for the Cu, Ga, and Mn sites but not for the Y site. Y_2_CuGaMn_4_O_12_ exhibits FM ordering at the B sites with an antiparallel arrangement of magnetic moments at the A′ or A″ site.

## Supporting Information Available

Additional SEM images and EDX results; structural parameters of Y_2_CuGaMn_4_O_12_ from synchrotron X-ray diffraction at 100, 297, and 700 K; Rietveld fits of neutron diffraction at 200 K; temperature dependence of the lattice parameters from neutron diffraction; temperature dependence of the lattice parameter for Au impurity; and numerical values of lattice parameters at different temperatures (PDF)
