# Supplementary material for: Triple A-Site Cation Ordering in the Ferrimagnetic Y2CuGaMn4O12 Perovskite
Source: Inorg Chem. 2022 Aug 31;61(36):14428–35. doi: 10.1021/acs.inorgchem.2c02343 (PMC9472281; doi:10.1021/acs.inorgchem.2c02343)
Supplement: Supplementary file 1 — ic2c02343_si_001.pdf [file ic2c02343_si_001.pdf]

Supporting Information (Online Material) for

Triple A-site Cation Ordering in the Ferrimagnetic $Y_2CuGaMn_4O_{12}$ Perovskite

Alexei A. Belik,[†] Dmitry D. Khalyavin,[*,§] Yoshitaka Matsushita,[#] and Kazunari Yamaura[†,‡]

[†] *International Center for Materials Nanoarchitectonics (WPI-MANA), National Institute for Materials Science (NIMS), Namiki 1-1, Tsukuba, Ibaraki 305-0044, Japan*

[§] *ISIS Facility, Rutherford Appleton Laboratory, Chilton, Didcot, OX11 0QX, United Kingdom, E-mail: dmitry.khalyavin@stfc.ac.uk*

[#] *National Institute for Materials Science (NIMS), Sengen 1-2-1, Tsukuba, Ibaraki 305-0047, Japan*

[‡] *Graduate School of Chemical Sciences and Engineering, Hokkaido University, North 10 West 8, Kita-ku, Sapporo, Hokkaido 060-0810, Japan*

Table S1. Structure Parameters of $Y_2CuGaMn_4O_{12}$ at T = 100 K, 297 K, and 700 K (at Ambient Pressure) from Synchrotron X-ray Powder Diffraction Data

T (K)	100	297	700
Source	Synchrotron X-ray (BL02B2 of SPring-8)		
Molecular weight (g/mol)	722.8258		
Wavelength (Å)	0.413854		
Measured d-space (Å)	0.328–11.401		
Used d-space (Å)	0.414–11.401		
Crystal system	tetragonal		
Space group	$P4_2/nmc$ (No. 137, cell choice 2)		
Z	2		
a (Å)	7.33101(2)	7.33884(2)	7.37866(3)
c (Å)	7.65747(4)	7.66251(4)	7.66318(4)
V (Å^3)	411.541(3)	412.693(3)	417.219(3)
z(Y)	0.22115(11)	0.22142(12)	0.22146(14)
B(Y) (Å^2)	0.542(14)	0.767(16)	1.226(23)
B(Cu-SQ) (Å^2)	0.79(7)	0.88(7)	1.21(8)
B(Ga-T) (Å^2)	0.57(6)	0.89(7)	1.38(8)
B(Mn-O) (Å^2)	0.357(15)	0.522(17)	0.823(23)
y(O1)	0.0678(6)	0.0677(6)	0.0669(6)
z(O1)	–0.0349(6)	–0.0353(6)	–0.0357(7)
B(O1) (Å^2)	0.70(10)	0.72(10)	0.81(12)
y(O2)	0.5448(6)	0.5442(6)	0.5436(7)
z(O2)	0.5929(7)	0.5928(7)	0.5921(7)
B(O2) (Å^2)	0.91(11)	1.21(12)	1.62(15)
x(O3)	0.4357(4)	0.4354(4)	0.4345(5)
B(O3) (Å^2)	1.04(11)	1.33(12)	2.09(15)
R_{wp} (%)	6.61	6.85	6.89
R_p (%)	5.14	5.30	5.35
R_I (%)	6.07	6.16	6.39

Y – 4d site (0.25, 0.25, z); Cu – 2a site (0.75, 0.25, 0.75); Ga – 2b site (0.75, 0.25, 0.25); Mn – 8e site (0, 0, 0); O1 and O2 – 8g site (0.25, y, z), and O3 – 8f site (x, –x, 0.25).
g(Y) = g(O1) = g(O2) = g(O3) = 1, were g is the occupation factor.
These g values were obtained from neutron diffraction at 200 K and fixed:
g = 0.798Cu+0.202Mn for the Cu site.
g = 0.800Ga+0.200Mn for the Ga site.
g = 0.8995Mn+0.0505Cu+0.05Ga for the Mn site.
SQ: square-planar; T: tetrahedral; O: octahedral.

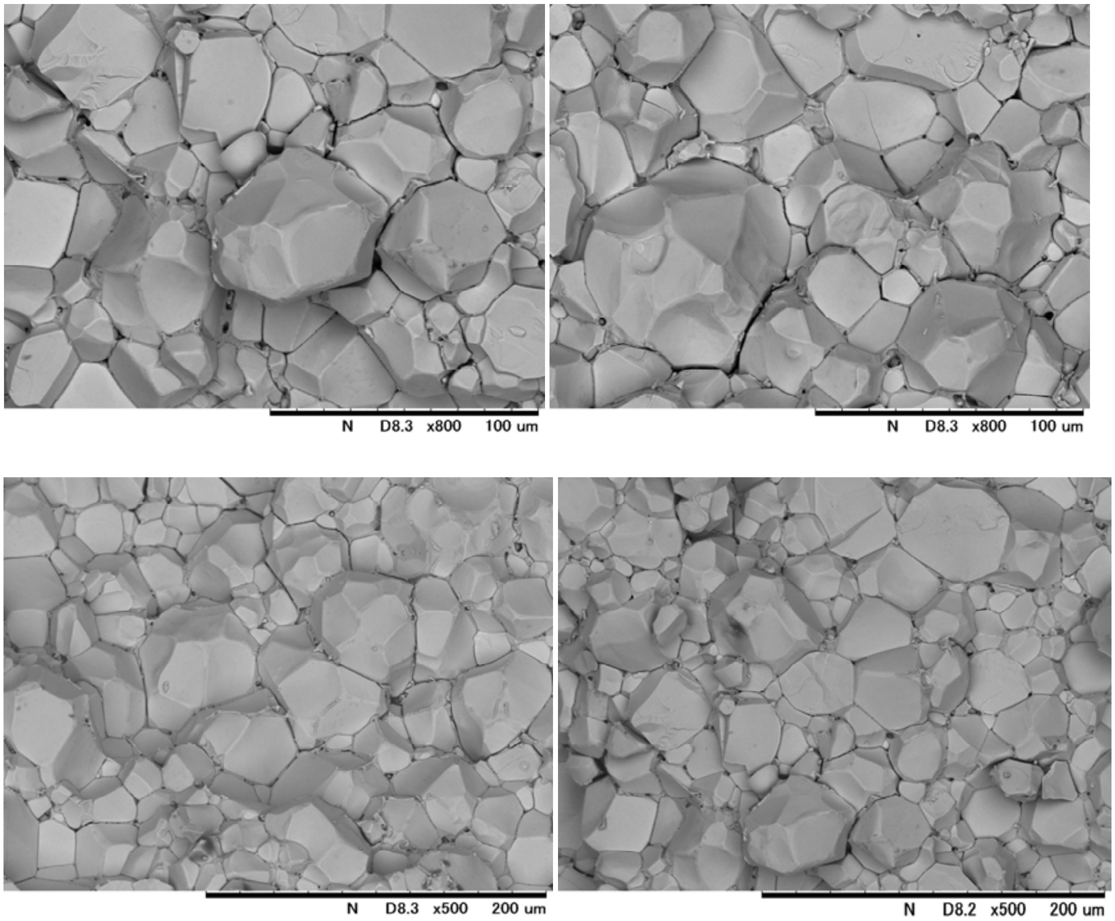

Figure S1a. Typical scanning electron microscopy (SEM) images of fractured surfaces of $Y_2CuGaMn_4O_{12}$. Typical crystallite sizes were 10–50 μm.

The starting mixture was:

$$4YMnO_3 + 2CuO + Ga_2O_3 + 1/0.339MnO_{1.839} + 0.178/0.339Mn_2O_3 = 2Y_2CuGaMn_4O_{12}$$

The starting mixture was reground in acetone 3 times and dried at 413 K for a few days.

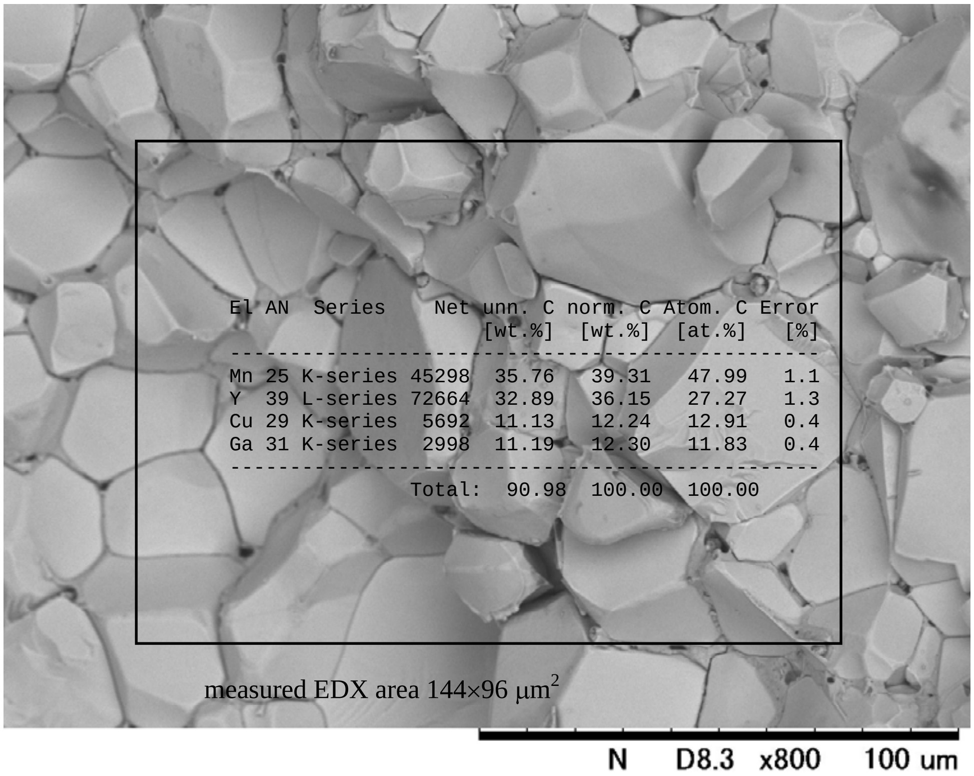

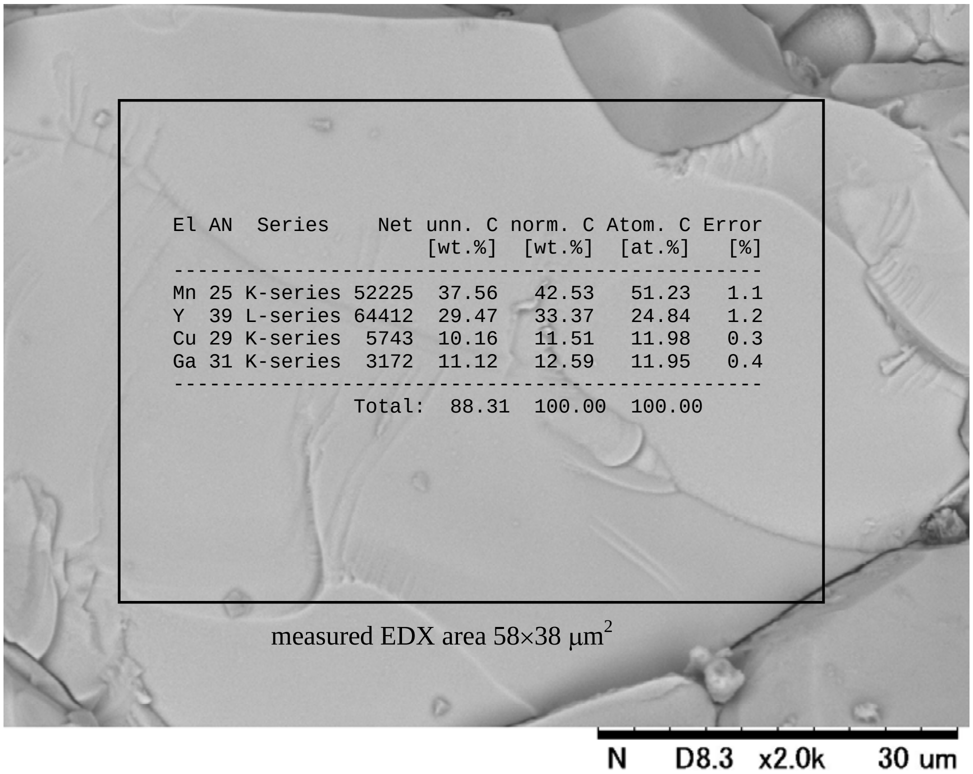

Figure S1b. Typical scanning electron microscopy (SEM) images of fractured surfaces of $Y_2CuGaMn_4O_{12}$ with EDX results. The black rectangles show scanned areas for EDX.

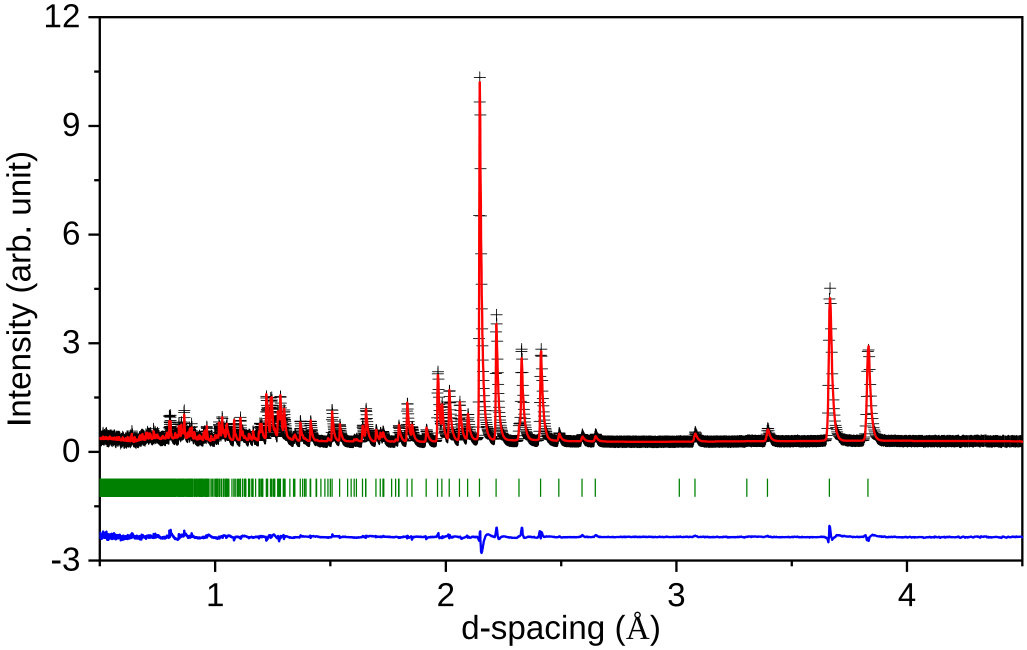

Figure S2. Experimental (black crosses), calculated (red line), and difference (blue line at the bottom) neutron powder diffraction patterns of $Y_2CuGaMn_4O_{12}$ at T = 200 K. The tick marks show possible Bragg reflection positions.

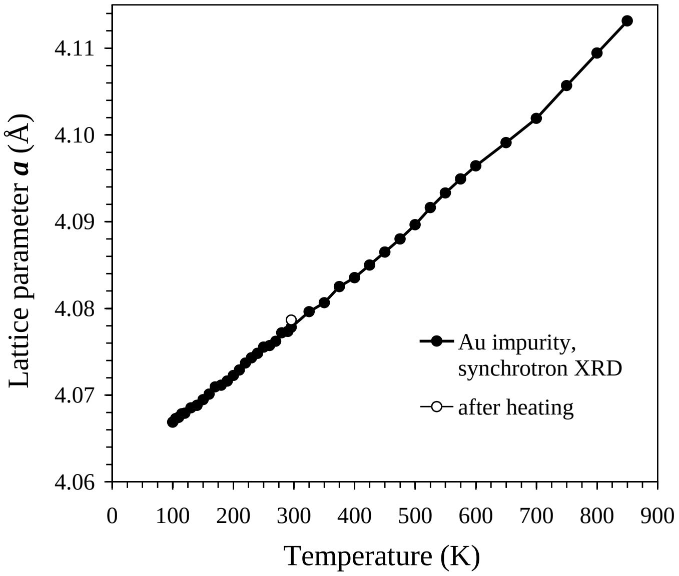

Figure S3. Temperature dependence of the *a* lattice parameter of the Au impurity in the $Y_2CuGaMn_4O_{12}$ sample from synchrotron X-ray powder diffraction data. The Au impurity can serve as an internal standard – no anomalies were observed suggesting reliable results for the temperature dependence of the main $Y_2CuGaMn_4O_{12}$ phase (reported in the main text). A white circle shows the lattice parameter at room temperature after the heating experiment.

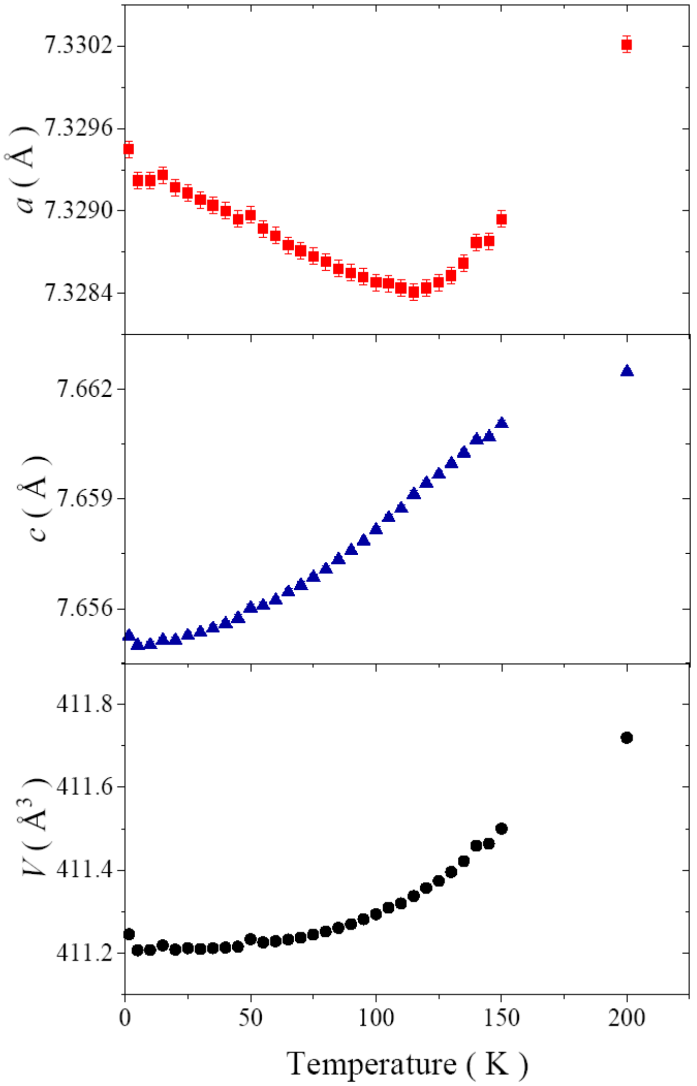

Figure S4. Temperature dependence of the lattice parameters in the $Y_2CuGaMn_4O_{12}$ sample from neutron powder diffraction data.

Table S2. Temperature Dependence of Lattice Parameters of $Y_2CuGaMn_4O_{12}$ from Neutron Powder Diffraction Data

T (K)	a (A)	a, error	c (A)	c, error	V (A3)	V, errror
1.5	7.32945	0.00006	7.65525	0.00010	411.246	0.007
5	7.32922	0.00006	7.65500	0.00010	411.207	0.007
10	7.32922	0.00006	7.65501	0.00010	411.208	0.007
15	7.32926	0.00006	7.65514	0.00010	411.219	0.007
20	7.32917	0.00006	7.65514	0.00010	411.209	0.007
25	7.32913	0.00006	7.65526	0.00010	411.212	0.007
30	7.32908	0.00006	7.65535	0.00010	411.210	0.007
35	7.32904	0.00006	7.65546	0.00010	411.212	0.007
40	7.32900	0.00006	7.65559	0.00010	411.214	0.007
45	7.32894	0.00006	7.65574	0.00010	411.216	0.007
50	7.32897	0.00006	7.65602	0.00010	411.234	0.007
55	7.32887	0.00006	7.65608	0.00010	411.226	0.007
60	7.32882	0.00006	7.65623	0.00010	411.229	0.007
65	7.32875	0.00006	7.65645	0.00010	411.233	0.007
70	7.32871	0.00006	7.65664	0.00010	411.238	0.007
75	7.32867	0.00006	7.65686	0.00010	411.245	0.007
80	7.32863	0.00006	7.65707	0.00010	411.252	0.007
85	7.32858	0.00006	7.65734	0.00010	411.261	0.007
90	7.32855	0.00006	7.65758	0.00010	411.270	0.007
95	7.32852	0.00006	7.65785	0.00010	411.282	0.007
100	7.32848	0.00006	7.65815	0.00010	411.294	0.007
105	7.32847	0.00006	7.65848	0.00010	411.310	0.007
110	7.32844	0.00006	7.65873	0.00010	411.320	0.007
115	7.32841	0.00006	7.65912	0.00010	411.338	0.007
120	7.32844	0.00006	7.65942	0.00010	411.357	0.007
125	7.32848	0.00006	7.65966	0.00010	411.374	0.007
130	7.32853	0.00006	7.65996	0.00010	411.396	0.007
135	7.32862	0.00006	7.66025	0.00010	411.422	0.007
140	7.32877	0.00006	7.66061	0.00010	411.459	0.007
145	7.32878	0.00006	7.66069	0.00010	411.464	0.007
150	7.32894	0.00006	7.66104	0.00010	411.500	0.007
200	7.33021	0.00006	7.66246	0.00010	411.719	0.007

Table S3. Temperature Dependence of Lattice Parameters of $Y_2CuGaMn_4O_{12}$ from Laboratory X-ray Powder Diffraction Data

T (K)	a (A)	c (A)	V (A3)
298	7.33884	7.66251	412.692
313	7.34008	7.66199	412.803
333	7.34161	7.66182	412.966
353	7.34315	7.66170	413.133
373	7.34486	7.66156	413.318
393	7.34645	7.66132	413.484
413	7.34816	7.66107	413.663
433	7.34983	7.66075	413.834
453	7.35144	7.66063	414.009
473	7.35316	7.66024	414.181
493	7.35487	7.66019	414.371
513	7.35639	7.66004	414.534
533	7.35802	7.65983	414.707
553	7.35961	7.65975	414.882
573	7.36120	7.65968	415.057
593	7.36268	7.65950	415.214
613	7.36420	7.65940	415.380
633	7.36580	7.65937	415.559
653	7.36728	7.65911	415.712
673	7.36886	7.65889	415.878
693	7.37036	7.65853	416.028
713	7.37181	7.65794	416.160
733	7.37347	7.65731	416.313
753	7.37535	7.65706	416.512
773	7.37696	7.65624	416.649
793	7.37869	7.65577	416.819
813	7.38043	7.65512	416.980
833	7.38218	7.65456	417.147
853	7.38400	7.65402	417.324
873	7.38576	7.65339	417.488
853	7.38493	7.65223	417.331
833	7.38399	7.65151	417.186
813	7.38271	7.65101	417.014
793	7.38151	7.65041	416.846
773	7.38031	7.65015	416.696
753	7.37889	7.64959	416.505
733	7.37765	7.64950	416.360
713	7.37623	7.64929	416.188
693	7.37479	7.64914	416.018
673	7.37479	7.64914	416.018
653	7.37191	7.64879	415.674
633	7.37046	7.64878	415.510
613	7.36898	7.64877	415.342
593	7.36740	7.64874	415.163
573	7.36588	7.64890	415.000
553	7.36430	7.64886	414.820

533	7.36268	7.64884	414.636
513	7.36113	7.64902	414.472
493	7.35950	7.64905	414.290
473	7.35788	7.64930	414.121
453	7.35617	7.64941	413.934
433	7.35456	7.64953	413.760
413	7.35294	7.64974	413.589
393	7.35137	7.64990	413.421
373	7.34975	7.64997	413.242
353	7.34822	7.65042	413.095
333	7.34663	7.65051	412.921
313	7.34514	7.65065	412.761
298	7.34396	7.65065	412.628

Table S4. Temperature Dependence of Lattice Parameters of $Y_2CuGaMn_4O_{12}$ from Synchrotron X-ray Powder Diffraction Data

measurement time (s)	T (K)	a (A)	c (A)	a (A) for Au	
300	100	7.33102	7.65747	4.06688	
10	105	7.33100	7.65782	4.06730	
10	110	7.33100	7.65832	4.06743	
10	115	7.33098	7.65865	4.06784	
10	120	7.33093	7.65875	4.06792	
10	130	7.33106	7.65940	4.06854	
10	140	7.33128	7.65983	4.06881	
10	150	7.33146	7.66045	4.06946	
10	160	7.33185	7.66079	4.07011	
10	170	7.33219	7.66117	4.07094	
10	180	7.33256	7.66149	4.07114	
10	190	7.33300	7.66178	4.07163	
10	200	7.33343	7.66200	4.07226	
10	210	7.33396	7.66230	4.07289	
10	220	7.33454	7.66259	4.07369	
10	230	7.33511	7.66271	4.07430	
10	240	7.33564	7.66295	4.07481	
10	250	7.33634	7.66303	4.07552	
10	260	7.33705	7.66313	4.07570	
10	270	7.33777	7.66322	4.07620	
10	280	7.33850	7.66329	4.07719	
10	290	7.33931	7.66336	4.07734	
10	297	7.33976	7.66348	4.07783	
10	325	7.34251	7.66344	4.07961	
10	350	7.34456	7.66329	4.08065	
10	375	7.34665	7.66301	4.08250	
10	400	7.34872	7.66268	4.08355	
10	425	7.35078	7.66242	4.08500	
10	450	7.35308	7.66207	4.08649	
10	475	7.35547	7.66174	4.08800	
10	500	7.35785	7.66137	4.08965	
10	525	7.36062	7.66119	4.09162	
10	550	7.36331	7.66097	4.09332	
10	575	7.36583	7.66084	4.09493	
10	600	7.36851	7.66094	4.09645	
10	650	7.37358	7.66214	4.09911	
300	700	7.37809	7.66248	4.10190	
10	750	7.38311	7.66148	4.10570	
10	800	7.38839	7.66174	4.10944	
10	850	7.39405	7.66241	4.11315	
10	297	7.34206	7.65969	4.07866	after heating to 850 K
300	297	7.33884	7.66251	4.07778	a separate run

Data in black were obtained in one run, that is, the sample was not moved from a measurement position. A separate run means that the sample was removed from a measurement position and inserted again. Therefore, a new centering procedure was performed, and a new center position was determined. This could result in different zero-shift parameters and different refined lattice parameters as a result.
